# Supplementary figures and images for: Therapeutic potentials of nonpeptidic V2R agonists for partial cNDI-causing V2R mutants
Source: PLoS One. 2024 May 15;19(5):e0303507. doi: 10.1371/journal.pone.0303507 (PMC11095762; doi:10.1371/journal.pone.0303507)

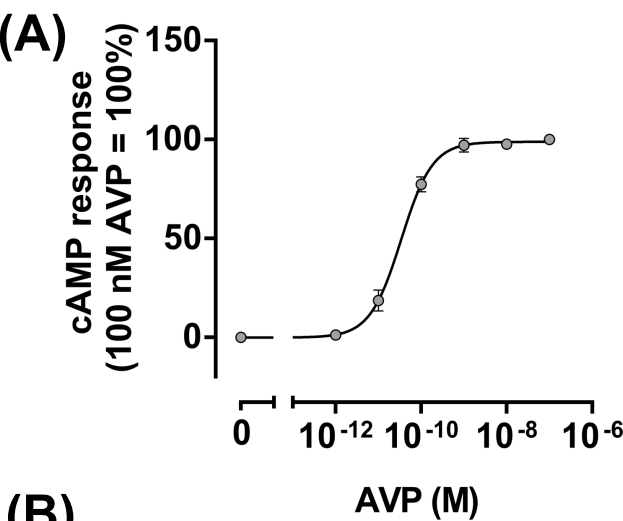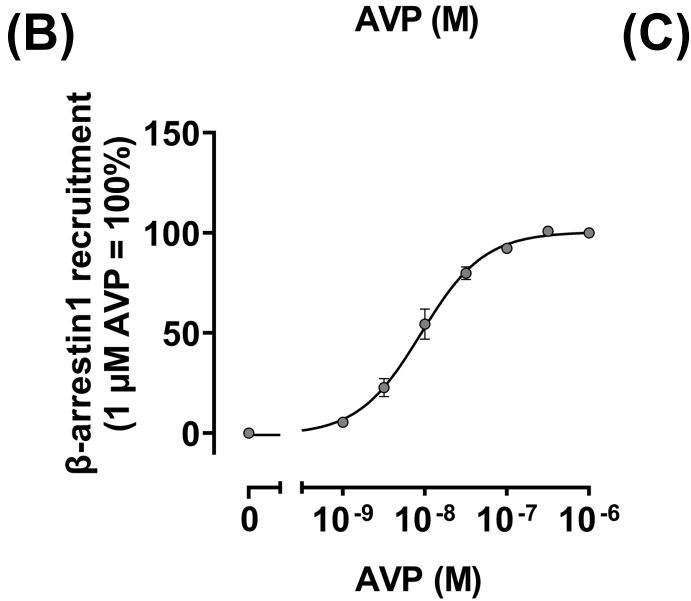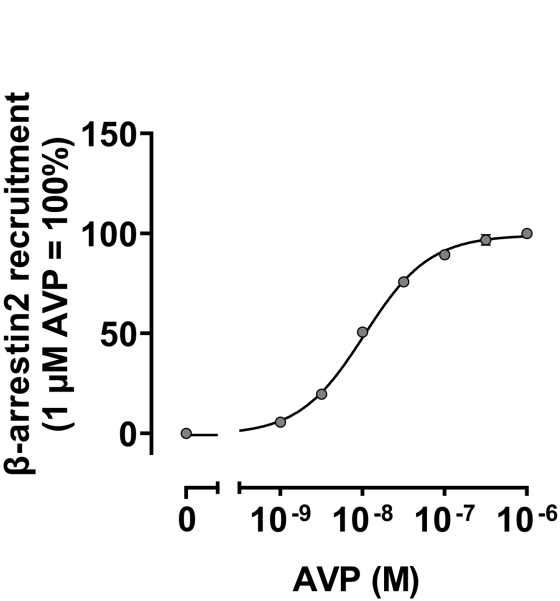

Supplement: S1 Fig — (A) Concentration-response curve for cAMP accumulation upon AVP stimulation. For each experiment, ligand-induced cAMP responses were normalized to the response induced by 100 nM AVP. Symbols and error bars are mean and SEM, respectively, of three independent experiments with each performed in duplicate. (B and C) Concentration-response curve for β-arrestin1 (B) and β-arrestin2 (C) recruitment upon AVP stimulation. For each experiment, ligand-induced cAMP responses were normalized to the response induced by 1 μM AVP. Symbols and error bars are mean and SEM, respectively, of three independent experiments with each performed in duplicate. (PDF) [file pone.0303507.s001.pdf]

Supplementary Figure 2

Kuramoto *et al.*

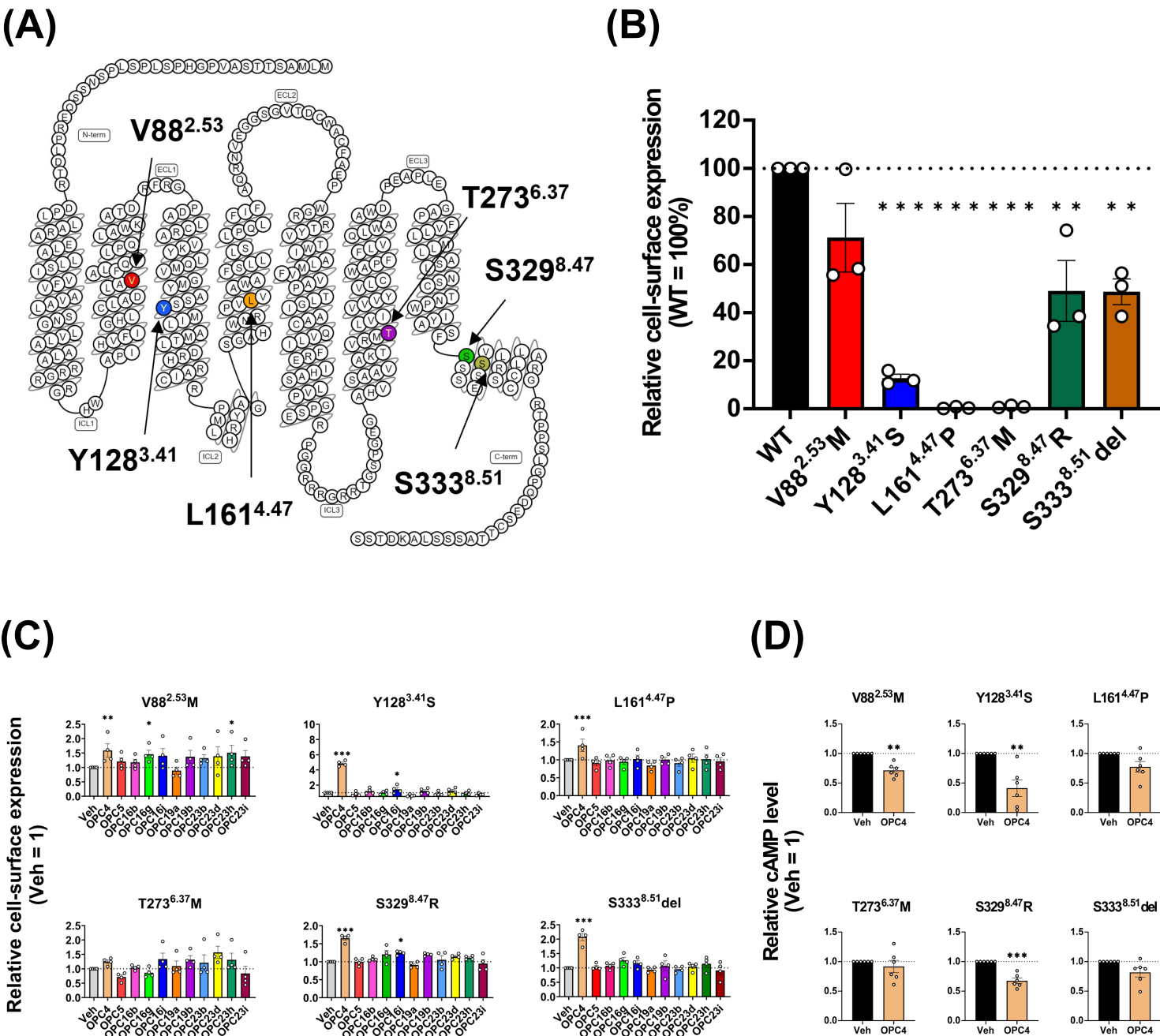

Supplement: S2 Fig — (A) Snake plot of human V2R. The residues altered in the mutants used in this study are colored in yellow. V882.53, Y1283.41, L1614.47, T2736.37, S3298.47 and S3338.51 are colored in red, blue, orange, purple, green and brown, respectively. (B) Measurement of cell-surface expression of wild-type and the six mutant V2Rs. For each experiment, cell-surface expression levels were normalized to the expression levels of wild-type V2R. Bars and error bars are mean and SEM, respectively, of three independent experiments with each performed in duplicate. *P < 0.05, **P < 0.01, ***P < 0.001 vs. WT. (C) Measurement of cell-surface expression of the six mutant V2Rs upon OPC analogues stimulation for 20 hours. For each experiment, cell-surface expression levels were normalized to the expression levels upon vehicle stimulation. Bars and error bars are mean and SEM, respectively, of four independent experiments with each performed in duplicate. For the statistical analyses, Data were analyzed by one-way ANOVA followed by the Dunnett’s test for multiple comparison analysis. *P < 0.05, **P < 0.01 vs. vehicle. (D) Measurement of cAMP levels of the six mutant V2Rs upon OPC4 stimulation for 20 hours. For each experiment, ligand-induced cAMP responses were normalized to the response induced by vehicle stimulation. Bars and error bars are mean and SEM, respectively, of six independent experiments with each performed in duplicate. Data were analyzed by unpaired t-test *P < 0.05, **P < 0.01, ***P < 0.001 vs. vehicle. (PDF) [file pone.0303507.s002.pdf]

Relative cAMP level  
(WT AVP = 100%)

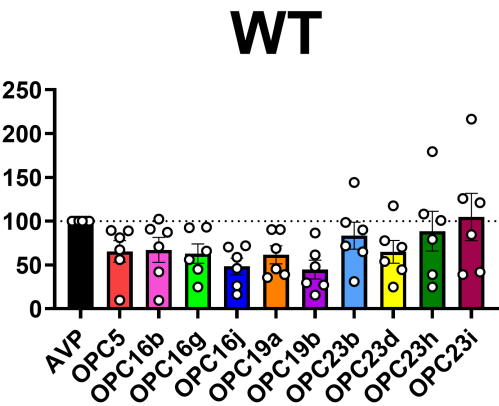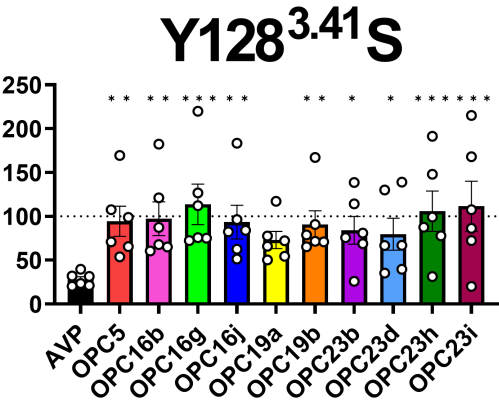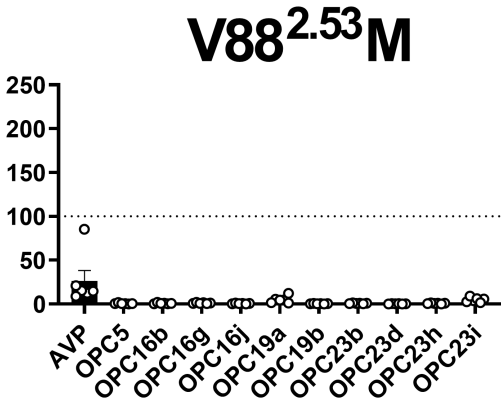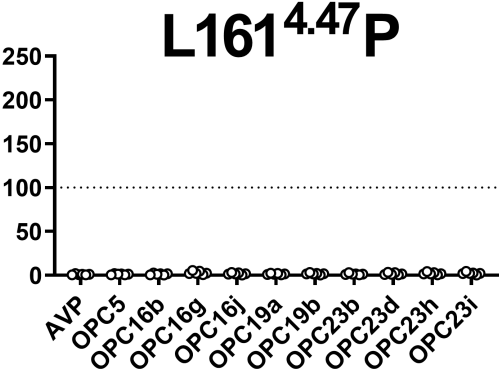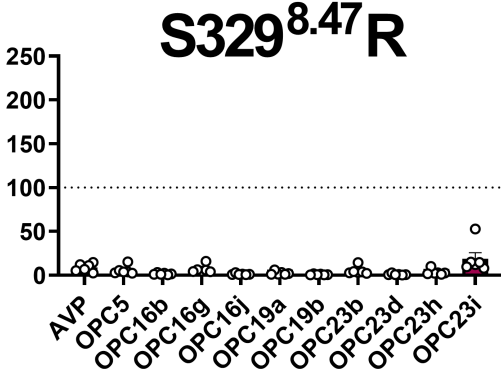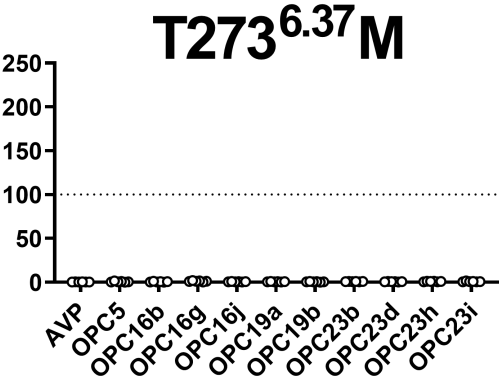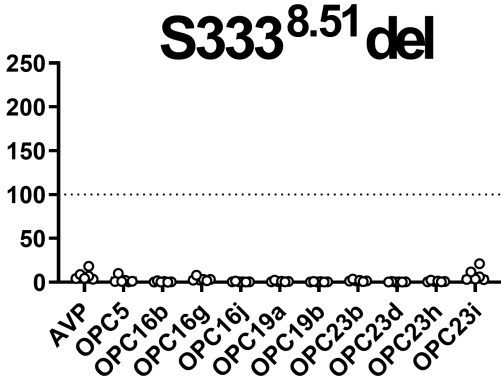

Supplement: S3 Fig — Measurement of cAMP levels of wild-type and the six mutant V2Rs upon AVP and OPC analogues stimulation for 20 hours. For each experiment, ligand-induced cAMP responses were normalized to the response induced by AVP at wild-type V2R. Bars and error bars are mean and SEM, respectively, of six independent experiments with each performed in duplicate. Data were analyzed by one-way ANOVA followed by the Dunnett’s test for multiple comparison analysis. *P < 0.05, **P < 0.01, *** P < 0.001 vs. AVP. (PDF) [file pone.0303507.s003.pdf]
