# Supplementary material for: Therapeutic potentials of nonpeptidic V2R agonists for partial cNDI-causing V2R mutants
Source: PLoS One. 2024 May 15;19(5):e0303507. doi: 10.1371/journal.pone.0303507 (PMC11095762; doi:10.1371/journal.pone.0303507)
Supplement: S1 Table — (PDF) [file pone.0303507.s004.pdf]

Supplementary Table 1

Kuramoto *et al.*

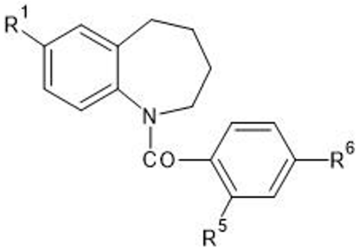

Compound	R^1	R^5	R^6
**OPC16b**	H	Cl	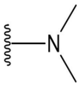
**OPC16g**	H	Cl	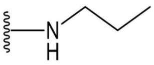
**OPC16j**	H	Cl	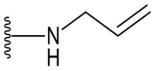
**OPC19a**	H	H	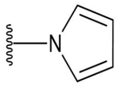
**OPC19b**	H	Cl	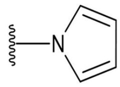
**OPC23b**	H	Cl	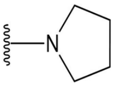
**OPC23d**	Cl	Cl	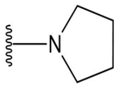
**OPC23h**	H	Cl	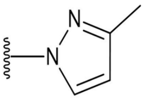
**OPC23i**	H	Cl	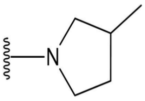
