# Supplementary material for: Therapeutic potentials of nonpeptidic V2R agonists for partial cNDI-causing V2R mutants
Source: PLoS One. 2024 May 15;19(5):e0303507. doi: 10.1371/journal.pone.0303507 (PMC11095762; doi:10.1371/journal.pone.0303507)
Supplement: S2 Table — (PDF) [file pone.0303507.s005.pdf]

Supplementary Table 2

Kuramoto *et al.*

Compound	pEC_{50} (cAMP response) (n = 3)	pEC_{50} (β-arrestin1 recruitment) (n = 3)	pEC_{50} (β-arrestin2 recruitment) (n = 3)
**AVP**	10.48 ± 0.09	8.05 ± 0.10	7.99 ± 0.01
**OPC5**	7.56 ± 0.23	6.85 ± 0.12	6.86 ± 0.19
**OPC16b**	7.76 ± 0.08	7.68 ± 0.51	ND
**OPC16g**	7.91 ± 0.06	6.88 ± 0.34	6.61 ± 0.45
**OPC16j**	7.59 ± 0.05	ND	ND
**OPC19a**	7.05 ± 0.06	ND	ND
**OPC19b**	7.13 ± 0.03	ND	ND
**OPC23b**	7.50 ± 0.01	ND	ND
**OPC23d**	7.06 ± 0.10	ND	6.51 ± 0.14
**OPC23h**	7.93 ± 0.10	ND	ND
**OPC23i**	8.05 ± 0.08	7.12 ± 0.06	7.07 ± 0.09
