# Supplementary material for: Therapeutic potentials of nonpeptidic V2R agonists for partial cNDI-causing V2R mutants
Source: PLoS One. 2024 May 15;19(5):e0303507. doi: 10.1371/journal.pone.0303507 (PMC11095762; doi:10.1371/journal.pone.0303507)
Supplement: Attachment — Submitted filename: Point-to-point response.pdf [file pone.0303507.s007.pdf]

Comment from the editor:

1. Please ensure that your manuscript meets PLOS ONE's style requirements, including those for file naming. The PLOS ONE style templates can be found at https://journals.plos.org/plosone/s/file?id=wjVg/PLOSOne_formatting_sample_main_body.pdf and https://journals.plos.org/plosone/s/file?id=ba62/PLOSOne_formatting_sample_title_authors_affiliations.pdf

We have verified that the manuscript meets the requirements.

2. Please note that PLOS ONE has specific guidelines on code sharing for submissions in which author-generated code underpins the findings in the manuscript. In these cases, all author-generated code must be made available without restrictions upon publication of the work. Please review our guidelines at https://journals.plos.org/plosone/s/materials-and-software-sharing#loc-sharing-code and ensure that your code is shared in a way that follows best practice and facilitates reproducibility and reuse.

Not applicable to the manuscript as it does not contain any author-generated code.

3. Thank you for stating the following financial disclosure: "A.I. was funded by KAKENHI JP21H04791, JP21H05113 and JP21H05037 from the Japan Society for the Promotion of Science (JSPS); JPMJFR215T and JPMJMS2023 from the Japan Science and Technology Agency (JST)."

Please state what role the funders took in the study. If the funders had no role, please state: "The funders had no role in study design, data collection and analysis, decision to publish, or preparation of the manuscript."

If this statement is not correct you must amend it as needed.

Please include this amended Role of Funder statement in your cover letter; we will change the online submission form on your behalf.

Since the funders had no role in our study, we have included the statement "*The funders had no role in study design, data collection and analysis, decision to publish, or preparation of the manuscript.*"

4. Thank you for stating the following in the Acknowledgments Section of your manuscript: "We thank Ayaki Saito and other members of the Inoue laboratory for critical reading and editing of the manuscript. We thank Dr. Hiroyuki Fujiki at Otsuka Pharmaceutical for the OPC5 agonists. A.I. was funded by KAKENHI JP21H04791, JP21H05113 and JP21H05037 from the Japan Society for the Promotion of Science (JSPS); JPMJFR215T and JPMJMS2023 from the Japan Science and Technology Agency (JST)."

We note that you have provided funding information that is not currently declared in your Funding Statement. However, funding information should not appear in the Acknowledgments section or other areas of your manuscript. We will only publish funding information present in the Funding Statement section of the online submission form.

Please remove any funding-related text from the manuscript and let us know how you would like to update your Funding Statement. Currently, your Funding Statement reads as follows: "A.I. was funded by KAKENHI

JP21H04791, JP21H05113 and JP21H05037 from the Japan Society for the Promotion of Science (JSPS); JPMJFR215T and JPMJMS2023 from the Japan Science and Technology Agency (JST)."

Please include your amended statements within your cover letter; we will change the online submission form on your behalf.

By following the instruction, we have removed the funding statement in the manuscript. Now, the Acknowlegement Section states as follows:
"We thank Ayaki Saito and other members of the Inoue laboratory for critical reading and editing of the manuscript. We thank Dr. Hiroyuki Fujiki at Otsuka Pharmaceutical for the OPC5 agonists.“

5. We note that your Data Availability Statement is currently as follows: "All relevant data are within the manuscript and its Supporting Information files."

Please confirm at this time whether or not your submission contains all raw data required to replicate the results of your study. Authors must share the "minimal data set" for their submission. PLOS defines the minimal data set to consist of the data required to replicate all study findings reported in the article, as well as related metadata and methods (https://journals.plos.org/plosone/s/data-availability#loc-minimal-data-set-definition).

For example, authors should submit the following data:

- The values behind the means, standard deviations and other measures reported;
- The values used to build graphs;
- The points extracted from images for analysis.

Authors do not need to submit their entire data set if only a portion of the data was used in the reported study.

If your submission does not contain these data, please either upload them as Supporting Information files or deposit them to a stable, public repository and provide us with the relevant URLs, DOIs, or accession numbers. For a list of recommended repositories, please see https://journals.plos.org/plosone/s/recommended-repositories.

If there are ethical or legal restrictions on sharing a de-identified data set, please explain them in detail (e.g., data contain potentially sensitive information, data are owned by a third-party organization, etc.) and who has imposed them (e.g., an ethics committee). Please also provide contact information for a data access committee, ethics committee, or other institutional body to which data requests may be sent. If data are owned by a third party, please indicate how others may request data access.

We prepared and submitted a excel file under the name of "source data" that contains all of the values used to build the graphs in the manuscript.

6. Please include captions for your Supporting Information files at the end of your manuscript, and update any in-text citations to match accordingly. Please see our Supporting Information guidelines for more information: http://journals.plos.org/plosone/s/supporting-information.

We have included captions for supporting tables and updated in-text citations accordingliy.

7. Please review your reference list to ensure that it is complete and correct. If you have cited papers that have been retracted, please include the rationale for doing so in the manuscript text, or remove these references and replace them with relevant current references. Any changes to the reference list should be mentioned in the rebuttal letter that accompanies your revised manuscript. If you need to cite a retracted article, indicate the article's retracted status in the References list and also include a citation and full reference for the retraction notice.

In the revised manuscript, we have added seven references (ref. 9-11, ref. 36-39) . There was no reference that was omitted from the originally submitted manuscript.

Comments to the Author

Reviewer #1:

Kuramoto et al investigate the effect of nonpeptidic V2R agonists on V2R mutants that cause congenital nephrogenic diabetes insipidus (cNDI) using NanoBiT and cAMP Glosensor assays. The authors show that the mutant proteins have impaired internalization and cAMP responses, and that the OPC5-related agonists are effective in elevating cAMP signalling in some mutants. While the study is generally well written and the findings will be of interest to clinicians and scientists investigating V2R and cNDI, the manuscript needs some amendments to improve clarity. These are all written changes (i.e. no additional experimental work). I have outlined these below.

Revisions

1. The methods are generally very well written with lots of detail. However, I couldn't see an explanation of how the data presented in the cAMP assays was derived. Presumably this was AUC, but this needs clarifying. This is important for Figure 3 where the authors state the cAMP levels are not affected at early time points but are at later time points. How does this correlate with Fig. 3A? Is the data in 3A reflective of 15 mins or 20 hours? I assume 15 mins (as it is likely AUC of the 30 minute assay), however this needs clarifying.

We thank the reviewer for pointing out the misleading parts. For both evaluation timepoints, we used the average of multiple measurements within a given time window. For the earlier time point, a time window during 13-15 minutes after ligand addition was selected for quantification as the response reached a plateau. At the later time points, luminescence values were measured 5 times over a 5-minute period after 20 hours of ligand treatment, and the average of these values was used. We have revised the relevant parts of the materials and methods section at lines 147-149 because the procedures were confusing and misleading.

2. A statistics section should be added to the methods as this is conventional in most publications.

We thank the reviewer for the comment. By following the reviewer's suggestion, we added a statistical analysis part in the materials and methods section.

3. The authors state that all mutants have a significant reduction in cell surface expression in Fig. S2. However, no stats are shown. Please add this to the figure.

We thank the reviewer for the comment. We added statistical information to Fig S2B. We confirmed that all mutants except V88$^{2.53}$M exhibit significant reduction of plasma membrane expression levels compared to wild-type V2R. Although V88$^{2.53}$M failed to show significant difference (p = 0.053), a trend toward reduced membrane expression was observed. Accordingly, we have corrected the manuscript to avoid confusion at lines 275-278 as follows:

> "*As a result, V2R mutants other than V88$^{2.53}$M exhibited reduced plasma membrane expression compared to wild-type V2R (Fig S2B). Although V88$^{2.53}$M failed to show significant differences (p = 0.053), a trend toward decreased membrane expression was observed.*"

4. There are several other figures where it is difficult to interpret data as no stats are shown (e.g. Fig. 2B, Fig. 3B-D). These should be added in.

We thank the reviewer for the suggestion. We added statistical information to Fig.2B and Fig. 3B-D.

In Fig. 2B, we modified the figure to demonstrate the feasibility of the HiBiT-based approach for quantifying expression levels. As a statistical analysis, we performed a linear regression analysis after log transformation of the data and calculated correlation coefficients. As a result, we observed a high degree of correlation (r^2 = 0.9382) and linearity over the range of our assay. We evaluated the expression levels of the mutants within this range of assay. We accordingly changed the configuration of the figure from a bar graph to an *x-y* plot. To convey the quantitative nature of the assay, we have corrected the manuscript at lines 261-263 as follows.
"The luminescence signals correlated with the amount of transfected plasmid, indicating the feasibility of the assay to evaluate plasma membrane expression levels (Fig 2B)."

In Fig. 3B, all mutants exhibited significantly smaller pEC_{50} values.

In Fig. 3C, the cAMP response by none of the V2R mutants was significantly different from the response by wild-type V2R. indicating that cAMP response levels immediately after AVP stimulation were not severely impaired in these mutants.

In Fig. 3D, the cAMP responses of all mutants (V88$^{2.53}$M, Y128$^{3.41}$S, L161$^{4.47}$P, T273$^{6.37}$M S329$^{8.47}$R, and S333$^{8.51}$del) were significantly lower than that of wild-type V2R, suggesting a weakened capacity of activating sustained cAMP responses.

We added statistical symbols to each figure to increase readability.

5. In Fig. S2C – in those mutants where the cell surface expression is increased, is this restored to similar levels to wild-type protein or not?

Plasma membrane expression levels of V88$^{2.53}$M, Y128$^{3.41}$S, S329$^{8.47}$R, and S333$^{8.51}$del mutants were restored to the same level as the wild type by OPC4 treatment (Fig R1). In the L161$^{4.47}$P mutant, where the plasma membrane expression level was low, OPC4 exhibited pharmacological chaperone activity, but that expression level was still much lower than that of the wild type. To clearly show the degree of recovery, we prepared a modified version of Fig. S2C with the vertical axis as Veh = 100% of WT as below (Fig. R1).

During the revision process we noticed an error in the statistical analysis of Fig S2C. The pharmacological chaperone activity of some OPC5 analogues was found to be significant, and thus changes were made to the main text accordingly (line 315-319).

> "*Among OPC5 analogues, OPC16g and OPC23h elevated plasma membrane expression of the V882.53M mutant, while OPC16j elevated plasma membrane expression of the Y1283.41S and S3298.47R mutants. Taken together, we observed that some OPC5 analogues exhibit pharmacological chaperone activity, but their effects were much smaller than those of OPC4.*"

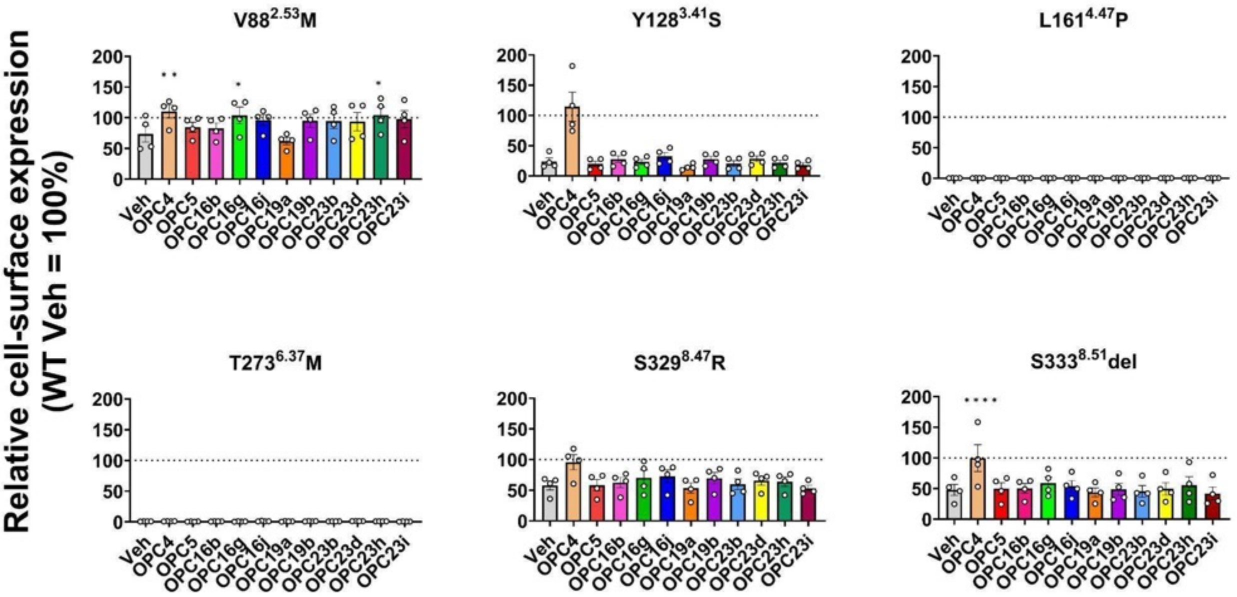

Fig. R1 Cell-surface expression of the six V2R mutants upon OPC5 analogue stimulation. Cell-surface expression levels of each V2R mutant under treatment of different compounds in Fig. S2C were normalized to those of the wild-type V2R with vehicle treatment.

6. Where is the data in Table S2 derived from. I initially thought Fig. 4, however, S2 has N=3 and Fig. 4 N=6. Please clarify this.

We thank the reviewer for pointing out the misleading part. Table S2 is derived from the data of Fig 1. We have added notations for Table S2 at line 236 and 241-242 in the main text.

7. Figure 4 would be improved by showing wild-type responses to determine whether the compounds restore cAMP levels to wild-type levels.

We thank the reviewer for the suggestion. We prepared a modified version of Fig. 4 (Fig. R2), in which the cAMP responses were normalized to those of the wild-type V2Rs upon AVP stimulation. As shown in Fig. S3 in the submitted manuscript, the cAMP levels of Y128$^{3.41}$S increased to levels comparable to wild-type cAMP upon OPC5 analogue stimulations (Fig. S3). The cAMP response levels of the V2R mutants other than the Y128$^{3.41}$S mutant are considerably lower than those of wild-type V2R, making it difficult to readily discern OPC5 analogues-induced effects. To make it easier to recognize the cAMP response level, we updated Fig. S3 in our revised manuscript.

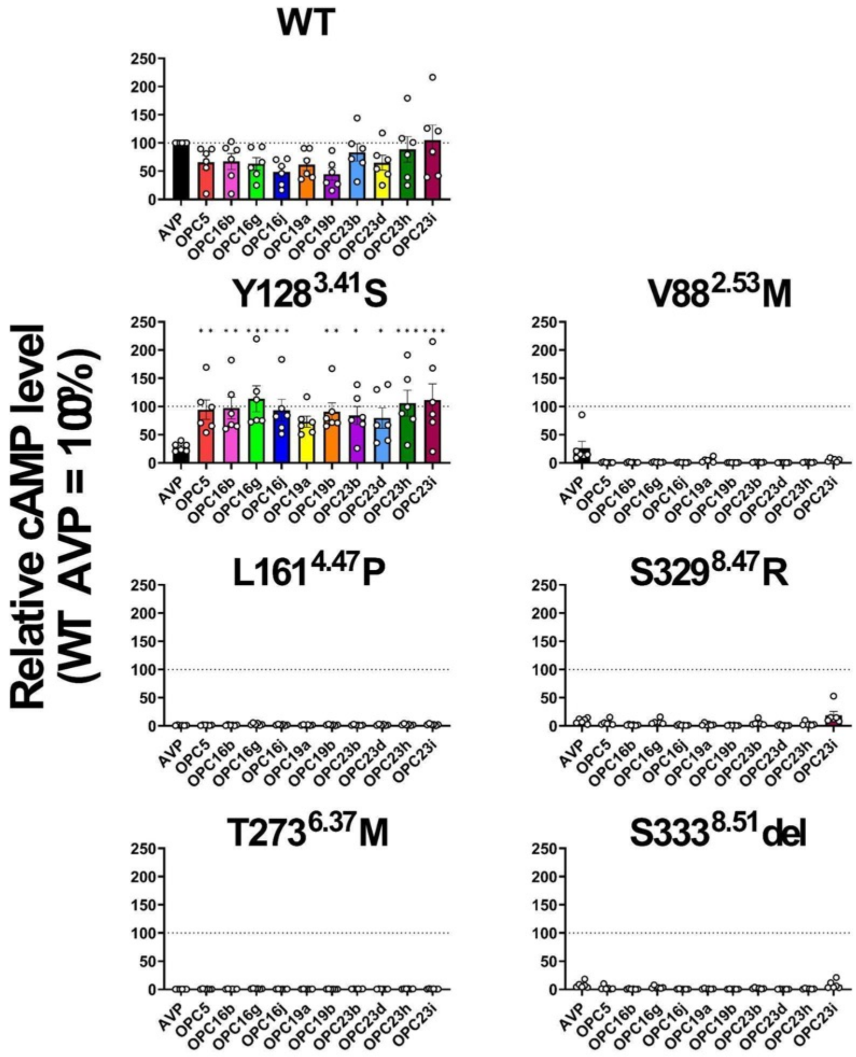

Fig. R2 Measurement of cAMP levels of wild-type and six V2R mutants upon AVP and OPC5 analogue stimulation.

cAMP levels in Fig. 4 were normalized to the wild-type V2R upon AVP stimulation.

8. The authors should clarify why they chose 20 hours as the time point to measure later signalling. Is AVP present physiologically for this time period?

The time point of 20 hours was used to match the time course of experiments measuring pharmacological chaperone activity and cAMP signaling activity. The pharmacological chaperone activity of V2R is frequently measured by treating the compound for 16 hours or longer to allow sufficient time for new receptors to be biosynthesized (Makita 2016, Mouillac 2014, Robben 2007). In this study, although pharmacological chaperone activity of OPC5 analogues were limited, we evaluated cAMP signaling activity at 20 hours in the cAMP assay in order to measure them under the influence of pharmacological chaperone activity.

We currently are unsure how much AVP and OPC5 analogues remain in the assay, as we do not determine the amount of them. Since the amount of peptidases such as trypsin involved in the degradation of AVP is thought to be low in the assay, the stability of AVP seems to be higher than its reported plasma half-life on the order of a few minutes. We assume that some amount of AVP is present throughout the assay, but the amount of AVP

in the culture supernatant will continuously decrease as it is metabolized by undergoing internalization along with V2R.

9. The authors briefly mention that there are two classes of effects on mutants in the results but then do not expand on this in greater detail in the Discussion. This should be discussed in the context of location of the mutants, degree of inactivation of signalling and other cNDI mutants (e.g. could you predict which mutants are likely to respond). This will make the manuscript of more interest to a wider readership.

We thank the reviewer for the suggestion. To provide insights into the effects of individual mutations in partial cNDI, we added one paragraph describing the classification and possible approach for individual mutations at line 393-407.

> "*Precision medicine has been an important strategy in the treatment of diseases whose pathophysiology is altered depending on mutations [37–39]. As mentioned earlier, the mechanisms that lead to functional loss of V2R can be divided into three categories. [3]. Consistent with previous reports [6,7,12], our study suggests that functional loss of the Y128$^{3.41}$S mutant is mainly due to defects in translocation from ER to the plasma membrane. Considering the efficacy of the OPC5 analogues for the Y128$^{3.41}$S mutant (Fig. 4 and S3), it is reasonable to assume that the OPC5 analogues may also effectively enhance cAMP signaling in other localization-defective mutants such as G201$^{5.34}$D [27]. In contrast, a loss of function of the S329$^{8.47}$R mutant is likely caused by defects in signaling function and may not be a promising target for our OPC5 analogue-based approach because the OPC5 analogues do not alleviate the signaling deficiency. In mutants with defective signaling activity, activation of G_s signaling pathway via a route other than V2R may serve as an effective strategy. Together, the OPC5 analogue-based approach may become beneficial to patients with partial cNDI caused by a certain type of V2R mutations, although further in-depth studies are required in future.*"

10. The authors should clarify why 100nM AVP was used in cAMP assays and 1uM in beta-arrestin assays.

We determined the AVP concentration used for normalization in each assay based on saturation of response. As shown in Fig. S1, the concentration of AVP at which the response saturates is different between the GloSensor-based cAMP assay and NanoBiT-β-arrestin recruitment assay. To normalize the OPC5 analogues-induced responses by the maximum responses upon AVP stimulation, we used different AVP concentrations as the reference. We added an explanation to the line 136 and 169 in the materials and methods section so that the criteria used to determine each concentration for normalization can be understood.

11. The authors state in the discussion that β-arrestin recruitment desensitises the receptor. Although this is the case for many GPCRs, AVPR is known to signal by β-arrestin over long periods of time (via endosomal signaling). The exposure of cells to ligand for 20 hours could affect this endosomal signaling too and this should be discussed in the manuscript.

We thank the reviewer for the comment. As the reviewer suggested, V2R has been known to trigger cAMP signaling from the endosome after being internalized. Since OPC5 analogues induce minimal β-arrestin activity, the OPC5 analogues seem to be less active in inducing endosomal signaling. Since the translocation of AQP2 to the plasma membrane is important for the treatment of cNDI, we added a description of the possible signal difference triggered by OPC5 analogues at line 384-389.

"*Although low β-arrestin activity can be advantageous of prolonged cAMP signaling for the OPC5 analogues, low β-arrestin activity also leads to reduced cAMP signaling originating from the endosome. Given that β-arrestin-mediated endosomal signaling is reported to contribute to the phosphorylation of AQP2 (Feinstein et al., 2013), further studies are needed to determine whether treatment with OPC5 analogues causes sufficient plasma membrane translocation of AQP2.*"

12. In all the figure legends need to add 'in' between 'performed' and 'duplicate' so that it reads 'each performed in duplicate.

We thank the reviewer for this comment. We added "in" to the appropriate position in all the figure legends.

13. Line 118 – add 'being' between 'before' and 'subjected'.

We thank the reviewer for this comment. We added "being" in the correct position at line 118.

Reviewer #2:

In this manuscript, the authors investigate the potential of OPC-51803 (OPC5) and related V2R agonists in addressing partial congenital nephrogenic diabetes insipidus (cNDI), a condition stemming from loss-of-function mutations in the type 2 vasopressin receptor (V2R). The OPC5-related ligands are shown to selectively activate the Gs-cAMP pathway without involving beta-arrestin1/2, suggesting a targeted approach in signaling. The manuscript details the response of six cNDI-related V2R mutants (V882.53M, Y1283.41S, L1614.47P, T2736.37M, S3298.47R, and S3338.51del) to OPC5-related agonists. The manuscript reports a moderate enhancement in cAMP responses in these mutants when treated with OPC5-related ligands compared to AVP, particularly under prolonged stimulation.

Overall the rationale for conducting this comprehensive study is sound. The methods and data analysis also appear to be sound. However, I have concerns about some experiment that seems to be missing and aspects of the experimental techniques used.

1. The authors should assess the activity of OPC analogues in assays directly downstream of Gs coupling with the receptor. This is because the current approach measures one aspect of receptor signaling in close proximity to the receptor (arrestin), while another is evaluated further downstream (cAMP). Such differential measurement depths can result in varying levels of signal amplification. It becomes particularly relevant given the manuscript assertions about biased signaling.

We agree with the reviewer's suggestion that it would be ideal to use an upstream G_s signaling assay to evaluate bias. We had attempted to assess the activity of the OPC analogues using a NanoBiT-based Gαs-Gβγ dissociation assay (Fig. R3). While we detected dissociation of the Gαs subunit by the OPC analogues, we faced difficulty of obtaining accurate assessment of their pharmacological activities owing to narrow signal window, moderate affinity of the OPC analogues and their non-specific effects on NanoBiT luminescence at high concentrations. Therefore, we employed the GloSensor-cAMP method, which is more sensitive to V2R activation, for assessment of G_s activation by the OPC analogues.
Due to the concerns about signal amplification, there could be inaccurate evaluations of the efficacy in the G_s signal. Since the β-arrestin signals were barely observed in our assays, unlike G_s signals, we have discussed the bias of the signal to some extent.

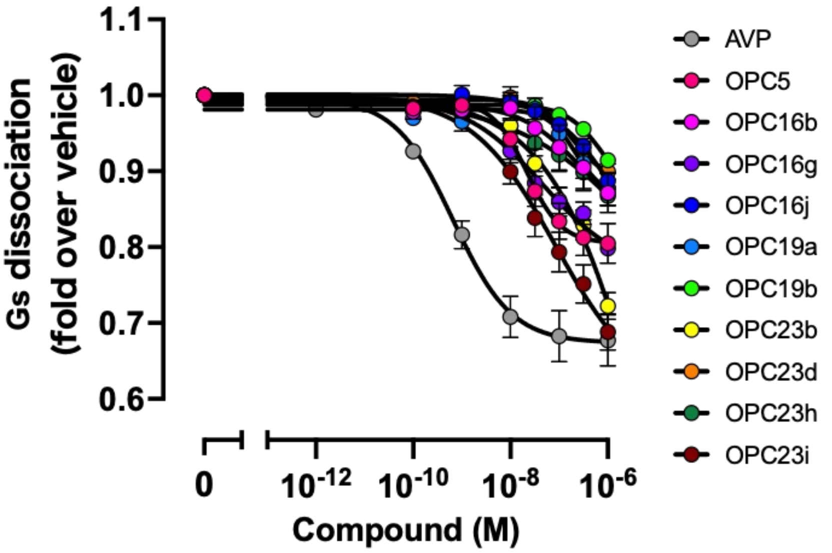

Fig. R3 Measurement of the dissociation of Gα-Gβγ by NanoBiT-based assay.
Ligand-induced dissociation of Gα-Gβγ was evaluated using the NanoBiT-based method. Gαs and $G\gamma_2$ were tagged with LgBiT and SmBiT, respectively, and expressed in the cells along with V2R, $G\beta_1$ and Ric8B. Luminescence signals measured during 10-15 min were averaged and used for evaluation. Bars and error bars are mean and SEM, respectively, of three independent experiments. Detailed methods were described previously (Inoue *et al.*, *Cell* (2019) 177, 1933–1947)

2. Please provide supplementary data using a cell-permeable cAMP analogue (e.g. 8-CPT) to ensure the Glosensor cAMP biosensor is not saturated at maximal ACP [authors' correction: AVP] ligand concentrations.

By following the Reviewer#2's suggestion, we used 8-CPT-cAMP to measure a maximum response and its range of response in the GloSensor-cAMP assay system. However, the kinetics data showed that response to 8-CPT-cAMP treatment, even at its saturating concentration (e.g., similar responses were obtained both from 100 µM and 1 mM conditions), were less than that of the AVP or forskolin (adenylyl cyclase activator) treatments for every time points, suggesting that the cAMP analogue does not adequately mimic the behavior of cAMP in the GloSensor-cAMP assay. Although not verified in this experiment, we consider that the GloSensor-cAMP assay is easily saturated because of signal amplification. In the revised manuscript, we added a concern of the high sensitivity of the GloSensor-cAMP assay at lines 245-247.

> "*Although the GloSensor-cAMP assay may not adequately assess efficacy of agonists due to its high sensitivity, these results suggest that the OPC5 analogues have a functional selectivity for the G_s-cAMP pathway over the β-arrestin pathway.*"

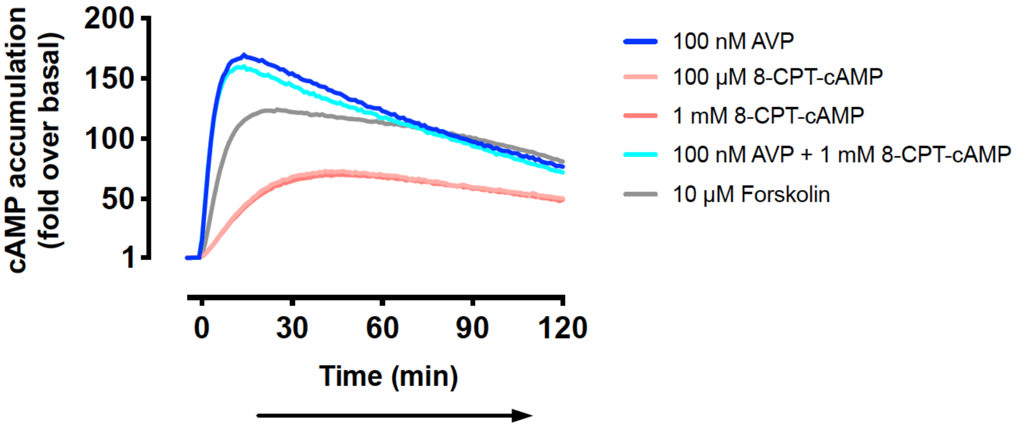

Fig. R4 cAMP accumulation kinetics in response to 8-CPT-cAMP.
HEK293A cells were transfected with plasmids encoding V2R and the GloSensor-22F reporter. Luminescence signals in response to ligand stimulation were measured for 120 min. Note that the response to 8-CPT-cAMP was slower than that to AVP stimulation and the response was not as strong as that to AVP. Representative data from two independent experiments are shown.

3. The authors should investigate the extended cAMP response both in the presence of OPC5 and during its washout (with or w/o antagonist), which could reveal important insights into the kinetics of OPC5, indicating its ability to induce enduring responses.

By following the Reviewer#2's suggestion, we performed washout experiments. In these experiments, conditioned media containing an OPC5 analogue was removed, and luminescence was continuously measured. However, we could not obtain meaningful results due to the intra- and inter-experiment variability. Alternatively, to fill the gap of being measured at 15 min and 20 h, we measured luminescence changes over a longer time period. We monitored the cAMP response to AVP stimulation and the OPC5 analogues for 2 h. Kinetics observed over 2 h did not differ significantly between AVP and OPC5 analogue stimulation. Therefore, we conclude that these ligands influence the V2R-Gs response in the later time point (after 2 h) rather than in the acute G_s response. We assume that the weaker receptor internalizing activity of OPC5 analogues than that of AVP (see new Fig. 2C in the revised manuscript) affects the cAMP response at a later time point.

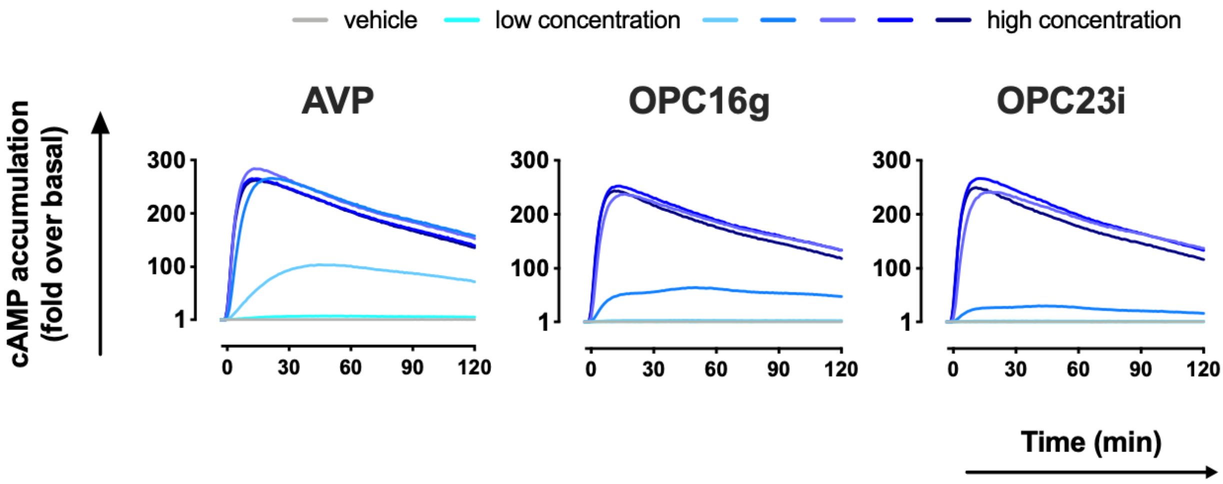

Fig. R5 Kinetics of cAMP accumulation responses upon AVP or OPC5 analogue stimulation.

HEK293A cells were transfected with plasmids encoding V2R and the GloSensor-22F reporter and evaluated for fold change in luminescence values in response to ligand stimulation. For either ligand stimulation, the cAMP response peaked before 20 min, and the signal slowly declined. The time course of the rise and fall of the signal did not differ significantly between ligands. Representative data from two independent experiments are shown.

4. The authors should test whether OPC5 analogues induce differential GRK phosphorylation, in alignment with their profile of biased agonism.

To the best of our knowledge, there are no reliable anti-phosphorylated V2R antibodies. Therefore, we are unable to directly verify this. As a complementary approach, we examined GRK dependence of β-arrestin recruitment using quadruple GRK2/3/5/6-deficient (ΔGRK2/3/5/6 or hereafter simply GRK-deficient) cells, which lack ubiquitous GRK subtypes. Furthermore, we also tested the effect of individual GRK subtypes on β-arrestin recruitment by re-expressing each GRK subtype in the GRK-deficient cells.
We found that AVP-induced β-arrestin recruitment was substantially attenuated in the GRK-deficient cells, confirming that β-arrestin recruitment is largely dependent on these four ubiquitous GRK subtypes. We note that the small β-arrestin recruitment of OPC23i in the parental cells was completely diminished in the GRK-deficient cells.
The re-expression experiment showed that overexpression of GRK2, GRK3, and GRK6 enhanced β-arrestin recruitment upon AVP stimulation, with GRK2 having the greatest effect. In contrast, the enhancement of β-arrestin recruitment by overexpression of GRK was observed but was weak upon OPC5 analogue stimulation. Notably, OPC5-induced β-arrestin recruitment was most enhanced with overexpression of GRK3, rather than GRK2, indicating that the GRK-subtype dependency varies depending on the ligand. Accordingly, we speculate that the differential phosphorylation pattern of V2R influences internalization and/or trafficking of V2R.

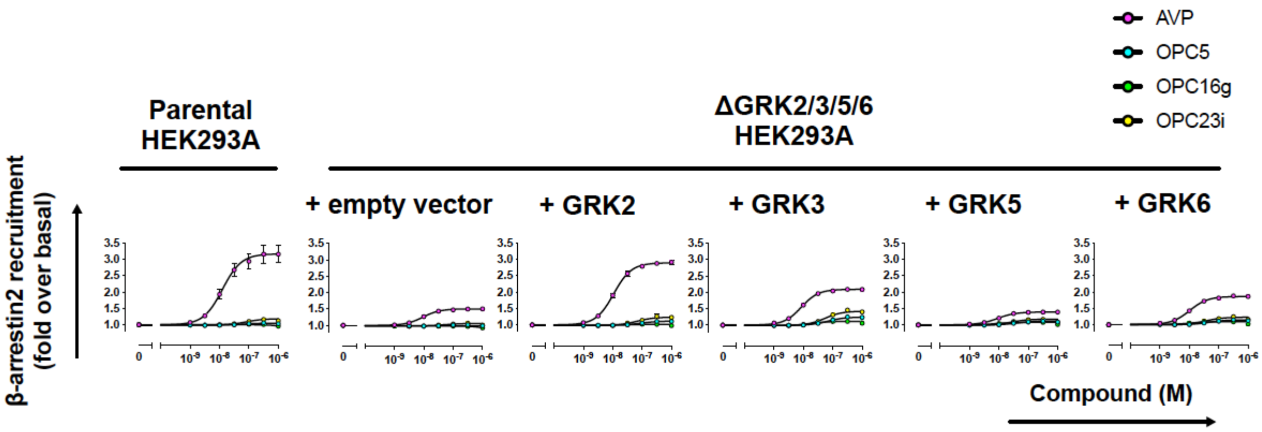

Fig. R6 GRK-subtype-dependent β-arrestin recruitment.
The parental HEK293A cells or the GRK2/3/5/6-deficient HEK293A cells were transfected with plasmids encoding the C-terminally SmBiT-fused V2R and the N-terminally LgBiT-fused β-arrestin2 constructs. As a GRK-subtype addback experiment, GRK2, GRK3, GRK5 or GRK6 was co-expressed together with the NanoBiT V2R and the β-arrestin2 constructs. The cells were then stimulated with titrated concentrations of AVP, OPC5, OPC16g or OPC23i and luminescent signals were measured as described in the method.Bars and error bars are mean and SEM, respectively, of three independent experiments.

5. The rationale behind measuring receptor internalization at 20 hours and any related observations on receptor recycling to the cell surface need clarification. Assessing total versus cell surface receptors can be achieved through cell lysis.

We thank the reviewer for the comments. Measurements at 20 hours after ligand stimulation were designed to evaluate the pharmacological chaperone activity of the compounds. We agree with the Reviewer#2 that measurements at shorter time course would lead to more definitive conclusions regarding receptor internalizing activity. Accordingly, we measured agonist-stimulated internalization of V2R within 30 min. We used the adenosine A3 receptor (with the N-terminal HiBiT fusion) as a negative control for the effect of V2R ligands. In addition to the parental HEK293A cells, we used β-arrestin1 and β-arrestin2 double-deficient HEK293A cells (Δ*ARRB1/2* cells) to answer questions in the comments from Reviewer#3 (point 1a and 1b). As expected, we observed a significant AVP-dependent internalization of V2R in the parental cells. In contrast, treatment of the OPC5 analogues induced negligible receptor internalization. We also observed that AVP-dependent receptor internalization was dependent on β-arrestin. Since these results better illustrate the receptor internalizing activity of the OPC5 analogues, we decided to include the data as a new Fig. 2C in the revised manuscript.

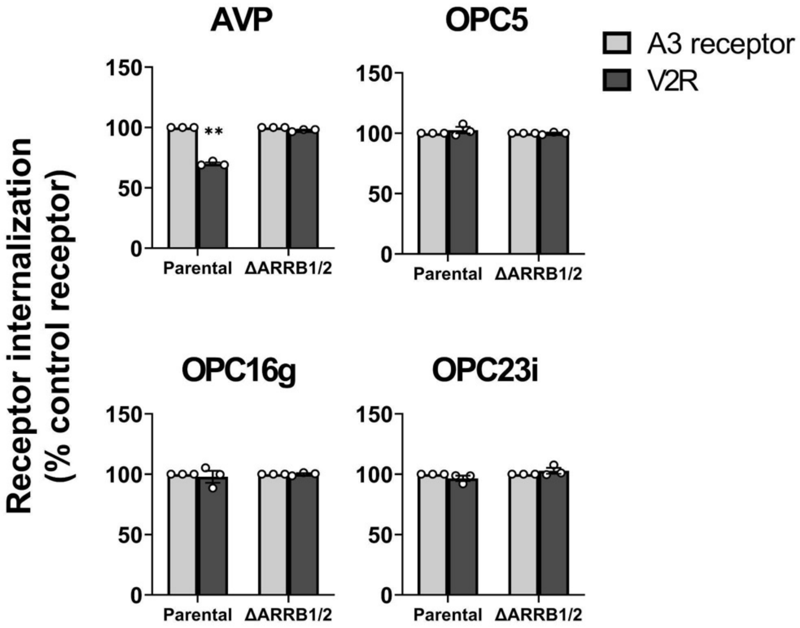

Fig. R7 (Fig. 2C in the revised manuscript) V2R agonist-dependent receptor internalization at early time points.
N-terminally HiBiT-tagged V2R or adenosine A3 receptor (control receptor) was expressed in cells and ligand-dependent changes in luminescence values were measured. Measured values during 20-30 min after ligand addition were averaged and used for evaluation. Each response was normalized to the control receptor. Bars and error bars are mean and SEM, respectively, of three independent experiments. *$P < 0.05$, **$P < 0.01$, ***$P < 0.001$.

6. Given that the authors observe cAMP stimulation effects at 20 hours, it'll be interesting for them to also examine the transcriptional profiling of Gs. Prolonged activation of the cAMP signaling pathway may lead to an amplification of downstream transcriptional responses mediated by Gs.

By following the Reviewer#2's suggestion, we evaluated the expression levels of the *GNAS* gene upon OPC5 analogue stimulation. As a result, we observed increased *GNAS* gene expression in several conditions, but the results varied depending on each OPC5 analogues and V2R mutants. The increase in *GNAS* mRNA expression was also observed in AVP treatment of L161$^{4.47}$P. Thus, it seems that upregulation of *GNAS* mRNA may be partly responsible for the increased cAMP response in prolonged OPC5 analogues treatment, but it would not be the only explanation.

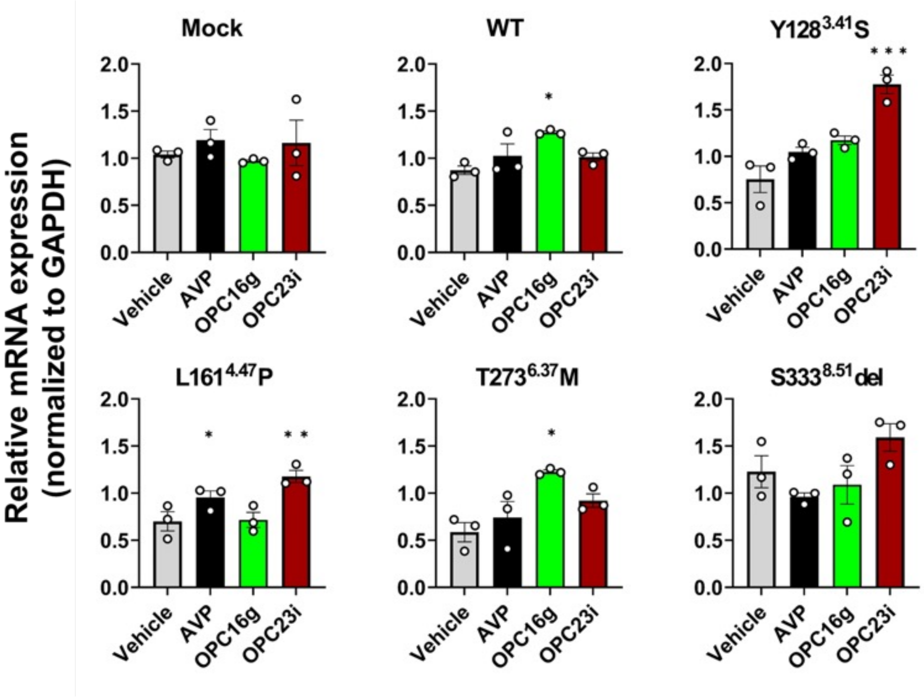

Fig. R8 *GNAS* expression upon AVP or OPC5 analogue simulation in V2R mutants.
HEK293A cells were transfected with plasmids encoding the wild-type V2R or the indicated V2R mutants. After compound treatment for 20 hours, the cells were lysed and mRNA was extracted.Expression levels of the *GNAS* gene relative to that of the GAPDH gene were quantified by a ΔCT method using a protocol described previously (Ono et al., 2023).Bars and error bars represent the mean and SEM, respectively, of three independent experiments. *$P < 0.05$, **$P < 0.01$, ***$P < 0.001$.

7. Noting that OPC4 increases receptor expression in some mutants, the ms should also assess its impact on cAMP signaling.

We prepared a graph of the cAMP response levels after 20 h OPC4 treatment, normalized to the cAMP response upon vehicle stimulation. Although it increased the cell-surface expression level of V2R mutants (Fig. 2C), treatment of OPC4 resulted in the decreased cAMP levels compared to the vehicle (Fig. S2D). This effect would be attributable to antagonistic properties of OPC4, which prevent V2R activation and consequently inhibit cAMP accumulation.
We added a description of cAMP levels after OPC4 treatment to the line 320-321 as follows.

> "*As expected, although it increased the cell-surface expression level of V2R mutants (Fig. S2C), treatment of OPC4 failed to induce cAMP accumulation (Fig. S2D)*"

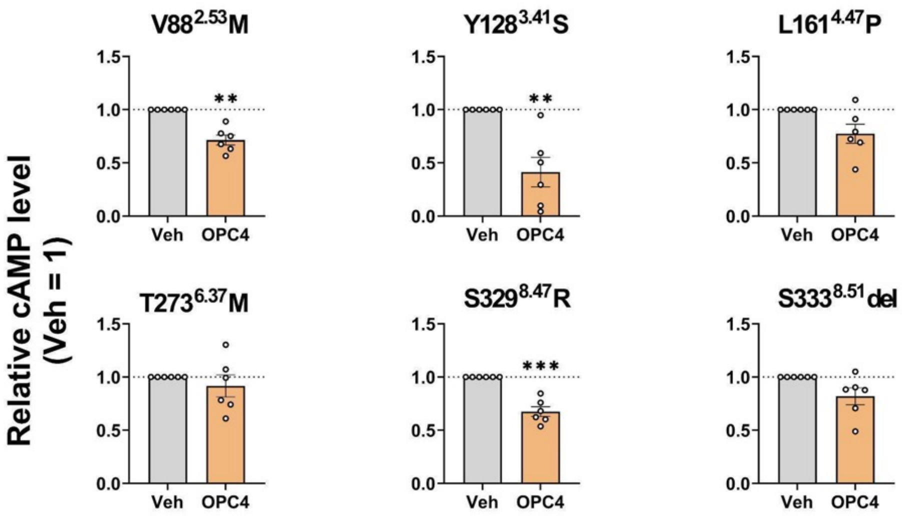

Fig. R9 (Fig. S2D in the revised manuscript) cAMP levels of the six V2R mutants upon OPC4 treatment for 20 hours.

HEK293A cells were transfected with each of the plasmids encoding the six V2R mutants, along with the cAMP GloSensor plasmid. Relative cAMP levels upon OPC4 for 20 hours were evaluated. Bars and error bars represent the mean and SEM, respectively, of six independent experiments. Each response was normalized to the response induced by the vehicle. $^{**}P < 0.01$, $^{***}P < 0.001$.

8. In the NanoBiT experiment, the authors have incubated cells with coelenterazine for 2 hours – how is the substrate not burning up and it becomes challenging to differentiate between kinetics of signaling versus kinetics of substrate.

In the NanoBiT assay, we utilized a sufficient amount of substrate to ensure the acquisition of robust data without substrate depletion during 2 hours incubation (Inoue et al., Cell (2019) 177, 1933–1947). In addition, the obtained signals are corrected by the vehicle stimulation, allowing us to distinguish between the kinetics of signaling and the kinetics of substrate.

9. Please comment on the activity of OPC5 analogues on the Gq pathway downstream of V2R and its physiological relevance to the disease.

We employed the TGFα shedding assay to assess activity of AVP and OPC5 analogues toward the $G_{q/11}$ pathway. The TGFα shedding assay is capable of detecting downstream of $G_{q/11}$ and $G_{12/13}$ pathways (Inoue *et al.*, *Nature Methods* (2012) 9,1021–1029; Inoue *et al.*, *Cell* (2019) 177, 1933–1947). To verify the contribution of the $G_{q/11}$ pathway in TGFα shedding response upon V2R activation, we used $G\alpha_q$ and $G\alpha_{11}$ double-deficient HEK293A (ΔGq/11) cells. We found that AVP stimulation elicited a robust response in the parental cells and that the response was almost completely silenced in the $\Delta G_{q/11}$ cells. In contrast, the OPC5 analogues did not elicit a response in the parental cells, suggesting that OPC5 analogues display negligible $G_{q/11}$ activation capacity. It is intriguing that OPC5 analogues show functional selectivity among G-protein subtypes; being capable of fully activating G_s signaling (Fig. 1B) while being poor for $G_{q/11}$ signaling. Thus, it is possible that these ligands have different physiological functions and roles.

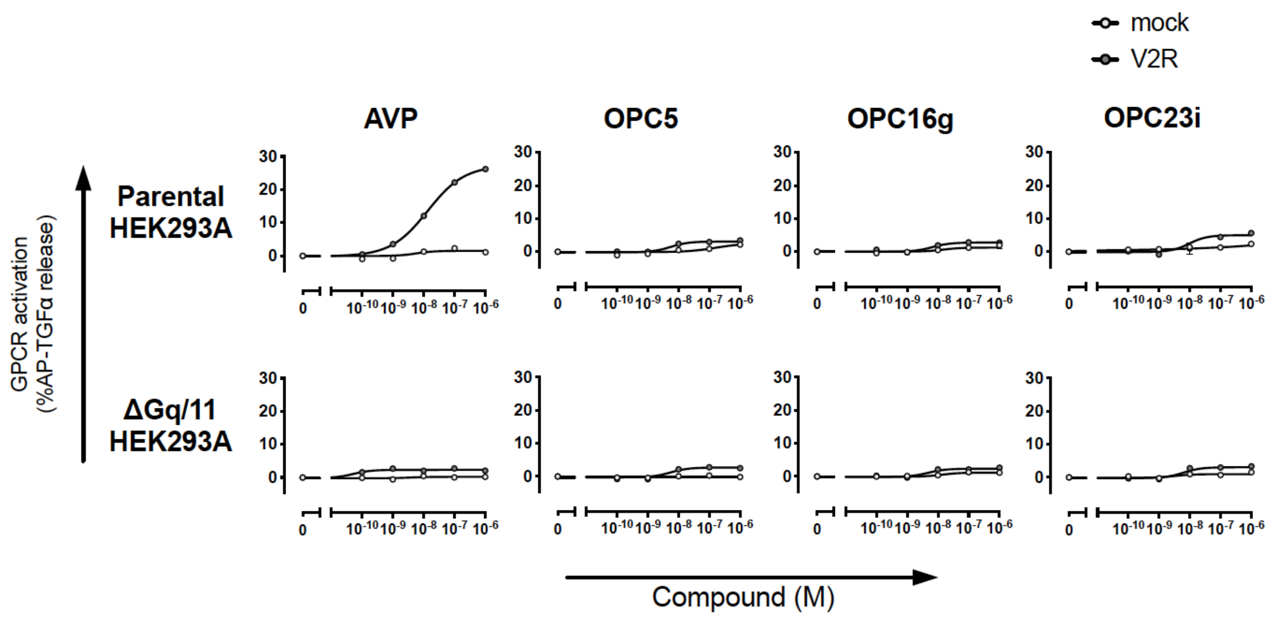

Fig. R10 Evaluation of $G_{q/11}$ pathway activation by TGFα shedding assay.
HEK293A cells were transfected with plasmids encoding the wild-type V2R and the AP-TGFα reporter. Release of AP to the cell supernatant in response to each ligand stimulation were evaluated by following the procedure described previously (Inoue et al., Nature Methods (2012) 9,1021–1029; Inoue et al., Cell (2019) 177, 1933–1947). The mock condition refers to conditions in which the V2R plasmid was omitted, but the AP-TGFα reporter was expressed. Bars and error bars are mean and SEM, respectively, of triplicate wells. Representative data from two independent experiments are shown.

Reviewer #3:

In this manuscript, Kuramoto et al. study the cAMP and β-arrestin 1/2 activity of a class of synthetic non-peptide agonists of the vasopressin V2 receptor (V2R). Using in-vitro signaling assays, they show that OPC5 and its analogs generate cAMP responses, albeit much lower than the native peptide agonist AVP, but do not recruit β-arrestin 1/2. Since some V2R mutants are implicated in partial congenital nephrogenic diabetes insipidus (cNDI), the authors compared receptor surface levels and cAMP production by OPC5 analogs in these mutants. Interestingly, OPC5 analogs improved cAMP responses from some of these mutants relative to AVP. These are exciting observations considering the potential for OPC5 analogs to be further developed as therapeutics for partial cNDI. Overall, the manuscript addresses an important question and opens several new directions of future investigation. I have a few suggestions and comments that could be addressed:

1. Fig 1C and D show that treatment with 1μM OPC5 (and its analogs) generate at the most ~10% arrestin recruitment. This assay was performed at a relatively early time point (15 min). On the other hand, receptor cell surface levels were measured after 20 hours of exposure to OPC5 analogs, which is interpreted as receptor internalization (Results, lines 245-246). In these data, there seems to be significant receptor internalization (relative to Veh) for most OPC5 analogs, although clearly not to the extent to which AVP induces internalization.

1a. Do OPC5 analogs induce similar internalization at the early with relatively acute exposure, especially considering previous data show that AVP causes rapid internalization of V2R (https://doi.org/10.1074/jbc.M112.445098; https://doi.org/10.1091/mbc.e16-12-0818; https://doi.org/10.7554/eLife.87754.3) ?

By following the Reviewer#3's suggestion, we evaluated receptor internalization activity of V2R agonists at an earlier time point. We used the adenosine A3 receptor as a control receptor to detect receptor-specific responses. We found that AVP stimulation resulted in significant receptor internalization within 30 min, but OPC5 analogues induced negligible receptor internalization. These results are consistent with the result that OPC5 analogues induce little β-arrestin recruitment in acute time points. Since these results better illustrate the receptor internalizing activity of the OPC5 analogues, we decided to include the data as a new Fig. 2C in the revised manuscript.

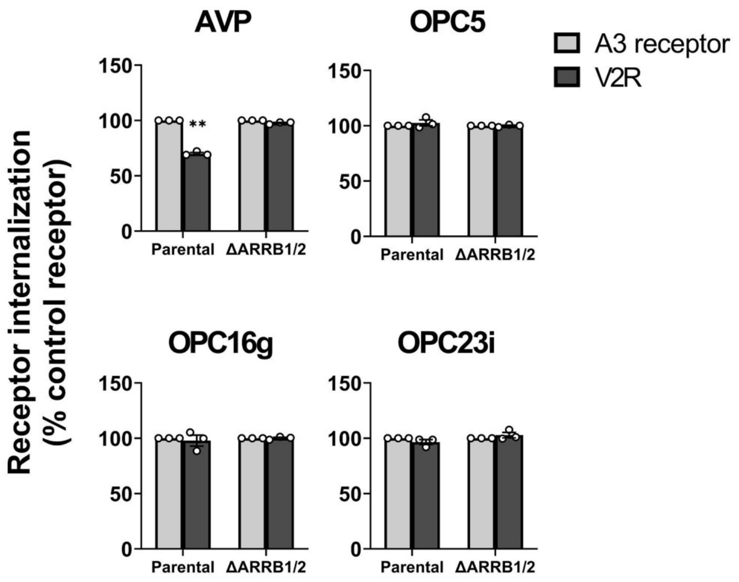

Fig. R11 (Same figure as Fig. R7; Fig. 2C in the revised manuscript) V2R agonist-dependent receptor internalization at early time points.

N-terminally HiBiT-tagged V2R or adenosine A3 receptor (control receptor) was transiently expressed in HEK293A cells and ligand-dependent changes in luminescence values were measured. Measured values during 20-30 min after ligand addition were averaged and used for evaluation. Each response was normalized to the control receptor. Bars and error bars are mean and SEM, respectively, of three independent experiments. $*P < 0.05$, $**P < 0.01$, $***P < 0.001$.

1b. Do the authors envision β-arrestin independent internalization with OPC5 analogs at the 20-hour time point?

In the experiments described above (Fig. R11), we tested the involvement of β-arrestin in V2R agonist-dependent receptor internalization using the β-arrestin1 and β-arrestin2 double-deficient HEK293A cells (Δ*ARRB1/2* cells). In the experiments with acute responses (Fig. R11), we found that agonist-induced receptor internalization was β-arrestin dependent. We postulate that OPC5 analogues gradually internalize the receptor over a long period of time via β-arrestin, or that activation of intracellular signaling down-regulates the amount of the receptor at the plasma membrane. However, we currently have no direct evidence to support either hypothesis.

1c. It might be a good idea to measure whole cell receptor populations to confirm if the reduction in surface receptor levels is due to endocytosis or overall downregulation of receptor levels.

We thank the reviewer for the suggestion. By following the Reviewer#3's suggestion, we performed HiBiT blotting to evaluate expression levels of V2R mutants at whole cell levels. After whole cell lysates were separated by SDS-PAGE and blotted onto membranes, we detected HiBiT-tagged V2R by treating the membranes with complementary LgBiT protein. As a result, we observed that the expression levels of V88$^{2.53}$M, Y128$^{3.41}$S, S329$^{8.47}$M and S333$^{8.51}$del were comparable to those of wild-type V2R at total protein levels, suggesting that these mutations caused defects in trafficking to the plasma membrane rather than protein synthesis. In contrast, we observed minimal expression of L161$^{4.47}$P and T273$^{6.37}$M at whole-cell levels.. This indicates that these mutations cause defects in the protein synthesis process and/or stability. Thus, this experiment indicates that, depending on the mutation, different pathways are involved in the reduction of plasma membrane expression levels.

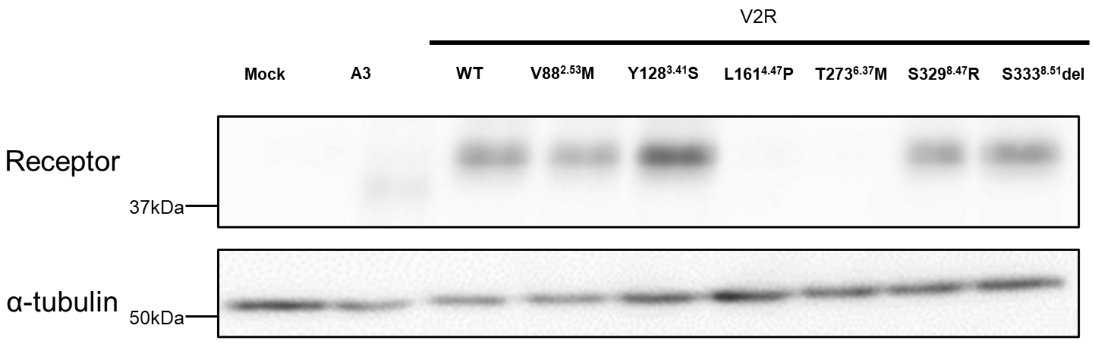

Fig. R12 Whole-cell expression levels of the V2R mutants.
N-terminally HiBiT-tagged V2R or adenosine A3 receptor (control receptor) was expressed in HEK293A cells and their expression was evaluated by the HiBiT-blot analysis. Cell lysates were separated by SDS-PAGE and blotted onto the nitrocellulose membrane. The membrane was incubated with LgBiT protein. After washout, the membrane was soaked with furimazine and the HiBiT-fused proteins were visualized by a chemiluminescent imager. α-tubulin was used as a loading control and their bands were visualized by a standard western blot procedure.

2. Fig S2B: Were the total receptor expression levels (plasma membrane + internal pool) comparable across mutants? Since these experiments were performed in HEK cells, it might be worth discussing on how plasma membrane localization of mutant receptors correlates with data available from endogenous systems.

Based on the HiBiT-blotting analysis (Fig. R12), the total receptor expression levels of V2R mutants were comparable to wild-type V2R except for the L161$^{4.47}$P and T273$^{6.37}$M. This indicated that the L161$^{4.47}$P and T273$^{6.37}$M cause abnormalities in the protein synthesis process and rapid degradation.

Employing MDCK cells is preferred for their ability to mimic physiological conditions such as apico-basal polarity in studying the membrane localization of V2R mutants. Currently, there are few examples of the mutants used in this study being evaluated in MDCK cells (Jean-Alphonse et al., 2006), making it difficult to recognize differences between cell types. However, it is worth noting that we observed decreased expression levels of V2R mutants and the increase in cell surface expression of the V2R mutants upon OPC4 stimulation in HEK293A cells, suggesting that HEK293A cells could also mimic physiological phenomena.

3. Discussion, line 366: "…non-peptidic agonists may provide a higher therapeutic effect than peptidic agonists on V2R mutants." Based on the results shown in Fig 4, this seems to be true only in case of Y128S, L161P and T273M mutants.

We thank the reviewer for the comments. As this sentence was an overstatement in the discussion, we corrected the phrase in the discussion at the line 389-392 as follows.
"Taken together, our results indicate that non-peptidic agonists could induce a higher cAMP response than peptidic agonists for partial cNDI-related V2R mutants with low plasma membrane expression levels, like the Y128$^{3.41}$S, L161$^{4.47}$P and T273$^{6.37}$M mutants."

4. The authors could consider including some more discussion on how specific OPC5 analogs could be potentially applied as biased agonists for cNDI-associated mutants.

We thank the reviewer for this comment. To provide insights into the effects of individual mutations in partial cNDI, we have added a discussion of partial cNDI-causing V2R mutants and the treatment according to the type of mutants based on differences in mechanism at the line 393-407.

> "*Precision medicine has been an important strategy in the treatment of diseases whose pathophysiology is altered depending on mutations [37–39]. As mentioned earlier, the mechanisms that lead to functional loss of V2R can be divided into three categories. [3]. Consistent with previous reports [6,7,12], our study suggests that functional loss of the Y128$^{3.41}$S mutant is mainly due to defects in translocation from ER to the plasma membrane. Considering the efficacy of the OPC5 analogues for the Y128$^{3.41}$S mutant (Fig. 4 and S3), it is reasonable to assume that the OPC5 analogues may also effectively enhance cAMP signaling in other localization-defective mutants such as G201$^{5.34}$D [27]. In contrast, a loss of function of the S329$^{8.47}$R mutant is likely caused by defects in signaling function and may not be a promising target for our OPC5 analogue-based approach because the OPC5 analogues do not alleviate the signaling deficiency. In mutants with defective signaling activity, activation of G_s signaling pathway via a route other than V2R may serve as an effective strategy. Together, the OPC5 analogue-based approach may become beneficial to patients with partial cNDI caused by a certain type of V2R mutations, although further in-depth studies are required in future.*"

*5. Placement of significance marks (*s): Fig S2C, S329R: Please check if the ** are correctly placed since OPC16j seems to show a higher cell surface expression relative to Veh, and not OPC19a.*

This error has been corrected. We thank the reviewer for pointing this out.

6. Discussion, line 361: please provide references for "greater stability over peptide ligands" for the OPC5 analogs.

We thank the reviewer for the comment. We decided to remove this statement because there was only unpublished data on the stability of the OPC5 analogues and it was difficult to properly compare them. We accordingly revised the manuscript at lines 379-381 as follows:
"*One of the possible reasons for the prolonged activation by the OPC5 analogues is their lower internalization activity.*"

7. Introduction: please provide references for V2R signaling described in lines 61-69.

We thank the reviewer for the comments. We added references about phosphorylation of AQP2 by V2R-Gs signaling and internalization of the receptor by V2R-β-arrestin at line 65 and 69.
